# Longitudinal Patterns of Care Transitions Among First-Time Nursing Home Residents: A Five-Year Study in Minnesota

**DOI:** 10.1093/geroni/igaf122.3030

**Published:** 2025-12-31

**Authors:** Dongjuan Xu, Emily Garcia, Greg Arling

**Affiliations:** Purdue University, West Lafayette, Indiana, United States; Purdue University, West Lafayette, Indiana, United States; Purdue University, West Lafayette, Indiana, United States

## Abstract

The objectives were to: 1) model the 5-year care trajectories of a cohort of first-time admissions to Minnesota nursing homes (NHs), while considering multiple outcomes of discharges to the community, NH readmission, acute hospitalizations, and mortality; and 2) determine the percentage of admissions and resident characteristics related to each trajectory. Using the Minimum Data Set and the Medicaid claims data files, we selected 11,943 residents, age 65+, admitted to Minnesota NHs in 2015 with no prior NH stays. Admissions were followed for 5 years (2015-2019) for survival and care transitions between NH, community, and acute hospitals. Latent class growth analysis was used to analyze longitudinal care trajectories. The optimal number of latent classes was four. Trajectory 1 (37%) had the shortest survival and highest initial (30-day) rate of rehospitalizations. Trajectory 2 (11%) had the second shortest survival and the highest hospitalization rate during years 4–5. These residents were initially discharged to the community but experienced a steadily increasing rate of NH readmission. Trajectory 3 (22%) demonstrated longer survival and the highest hospitalization rate during years 1-3. Trajectory 4 (30%) experienced the most successful transitions to the community and the best long-term outcomes, characterized by the fewest NH readmissions, longest survival, and lowest hospitalization rates. Our findings highlight the heterogeneity of outcomes for the NH population. The trajectories were distinguished by age, dementia, ADL dependency, and comorbid conditions. Understanding this heterogeneity is crucial for developing appropriate care models and policies to meet the needs of an increasingly diverse NH population.

